# Coagulation profile of human COVID-19 convalescent plasma

**DOI:** 10.1038/s41598-021-04670-1

**Published:** 2022-01-12

**Authors:** Allan M. Klompas, Noud van Helmond, Justin E. Juskewitch, Rajiv K. Pruthi, Matthew A. Sexton, Juan C. Diaz Soto, Stephen A. Klassen, Katherine A. Senese, Camille M. van Buskirk, Jeffrey L. Winters, James R. Stubbs, Scott A. Hammel, Michael J. Joyner, Jonathon W. Senefeld

**Affiliations:** 1 Department of Anesthesiology and Perioperative Medicine, Mayo Clinic, 200 First Street SW, Rochester, MN 55905 USA; 2 Department of Anesthesiology, Cooper Medical School of Rowan University, Cooper University Health Care, Camden, NJ USA; 3 Department of Laboratory Medicine and Pathology, Mayo Clinic, Rochester, MN USA; 4 Division of Hematology, Department of Laboratory Medicine and Pathology, Mayo Clinic, Rochester, MN USA

**Keywords:** Coagulation system, Infectious diseases

## Abstract

Convalescent plasma is used to treat COVID-19. There are theoretical concerns about the impact of pro-coagulant factors in convalescent plasma on the coagulation cascade particularly among patients with severe COVID-19. The aim of this study was to evaluate the coagulation profile of COVID-19 convalescent plasma. Clotting times and coagulation factor assays were compared between fresh frozen plasma, COVID-19 convalescent plasma, and pathogen-reduced COVID-19 convalescent plasma. Measurements included prothrombin time, activated partial thromboplastin time, thrombin time, fibrinogen, D-dimer, von Willebrand factor activity, von Willebrand factor antigen, coagulation factors II, V, VII–XII, protein S activity, protein C antigen, and alpha-2 plasmin inhibitor. Clotting times and coagulation factor assays were not different between COVID-19 convalescent plasma and fresh frozen plasma, except for protein C antigen. When compared to fresh frozen plasma and regular convalescent plasma, pathogen reduction treatment increased activated partial thromboplastin time and thrombin time, while reducing fibrinogen, coagulation factor II, V, VIII, IX, X, XI, XII, protein S activity, and alpha-2 plasmin inhibitor. The coagulation profiles of human COVID-19 convalescent plasma and standard fresh frozen plasma are not different. Pathogen reduced COVID-19 convalescent plasma is associated with reduction of coagulation factors and a slight prolongation of coagulation times, as anticipated. A key limitation of the study is that the COVID-19 disease course of the convalesced donors was not characterized.

## Introduction

Passive immunotherapy has been used since the late nineteenth century, and in 1901, the first Nobel Prize in Physiology or Medicine was awarded for serum therapy for patients with diphtheria^1^. The coronavirus disease 2019 (COVID-19) pandemic renewed interest in passive immunotherapy and involved the widespread use of convalescent plasma to treat patients with COVID-19. Although there is controversy about the potential therapeutic effects of convalescent plasma in the context of COVID-19, there is clear, substantial evidence supporting the safety profile of convalescent plasma^2,3^. Despite the reports of safety, there are theoretical concerns about the impact of pro-coagulant factors in convalescent plasma on the coagulation cascade particularly among patients with severe COVID-19 receiving convalescent plasma transfusions^4^.

Fresh frozen plasma contains physiological ratios of both pro- and anti-coagulant proteins^5^. Clinical studies have shown that COVID-19 may predispose patients to thrombotic disease^6,7^. Multiple studies have linked elevations of D-dimer, fibrinogen, and factor VIII to severe COVID-19^8–11^, and other coagulation factors may play a role as well^12^. Theoretically, plasma from a convalesced COVID-19 patient could contain more procoagulant or less anticoagulant proteins than regular fresh frozen plasma, and when transfused to a current COVID-19 patient contribute to risk of thrombosis.

Thus, our primary purpose was to evaluate the coagulation profile of COVID-19 convalescent plasma in comparison to standard fresh frozen plasma. We hypothesized that the coagulation profile of COVID-19 convalescent plasma would not be different from fresh frozen plasma.

Additionally, we assessed the coagulation profile of pathogen-reduced COVID-19 convalescent plasma as a secondary aim. The emergence of the novel pathogen that causes COVID-19, severe acute respiratory syndrome coronavirus 2 (SARS-CoV-2), increased interest in the use of existing pathogen reduction technologies for blood transfusion services. Psoralen-UV based pathogen reduction of plasma is now increasingly used by blood banks to inactivate potential pathogens, including viruses, bacteria, protozoa, and leukocytes contaminating the plasma; however, the effect of pathogen reduction on the coagulation profile of COVID-19 convalescent plasma is unknown. We hypothesized that the pathogen reduction process would be associated with loss of coagulation factor activity, as observed previously^13,14^.

## Methods

### Sources of human plasma

The Mayo Clinic institutional review board approved this study (IRB 20-012646) and waived the requirement to obtain informed consent. All blood donors provided permission to the blood center to use de-identified donor information and plasma samples for medical research. This research adhered to the requirements set forth in the declaration of Helsinki^15^. All plasma donations were performed at the FDA-registered and AABB-accredited Mayo Clinic Blood Donor Center in Rochester, Minnesota, USA. All plasma was collected by an apheresis collection technique using the Trima Accel Multiplasma Set (Terumo BCT, Lakewood, CO, USA). Forty-five plasma units from plasma donations were included in the research, including 15 fresh frozen plasma donations from healthy adult volunteers and 30 plasma donations from adults who had recovered from COVID-19 (Table 1). The criteria for individuals to be eligible to donate convalescent plasma included a history of laboratory confirmed COVID-19 and being at least 14 days after resolution of symptoms. Fifteen out of the 30 convalescent plasma donations analyzed in this study were convalescent plasma donations that had undergone pathogen reduction. All 45 samples were stored at −34 °C until the point of testing when they were thawed via standard operating procedures.

**Table 1:** Plasma donor demographics and sample storage characteristics.

Variable	Convalescent plasma	Pathogen-reduced convalescent plasma	Fresh frozen plasma	*p* values
No. of males	7	7	9	–
No. of females	8	8	6	–
Total no	15	15	15	–
Age (years)	46.2 ± 13.0	52.4 ± 14.6	46.5 ± 17.0	^ns,^ ^ns,^ ^ns^
Time spent frozen (days)	267 ± 61	194 ± 72	28 ± 13	***^,^ ***^,^ ***
Time since prior COVID + test (days)	64 ± 40	75 ± 37	–	^ns^
Time since COVID symptom resolution (days)	60 ± 36	72 ± 40	–	^ns^

Data are reported as count or mean ± standard deviation (SD). Data are compared using one-way analysis of variance with Tukey’s honestly significant difference (HSD) post hoc test to determine *p* values. *p* values (ns, *p* > 0.05; *p < 0.05; **p < 0.01; and ***p < 0.001) are reported in the order of the following comparisons: convalescent plasma to pathogen-reduced convalescent plasma, convalescent plasma to fresh frozen plasma, and pathogen-reduced convalescent plasma to fresh frozen plasma.

### Laboratory assays

The manufacturers’ instructions were followed for all assays, and analyses were performed on the ACL TOP 700 Instruments (Instrumentation Laboratory, Bedford, MA, USA). All testing kits were obtained from Instrumentation Laboratory. The prothrombin time (PT) and one-stage PT based assays for coagulation factors VII, X, V and II were performed using Recombiplastin 2G. The activated partial thromboplastin time (aPTT) assay and one-stage aPTT based assays for coagulation factors XII, XI, IX and VIII were performed using SynthASil. Three dilutions (1:10, 1:20, and 1:40) were automatically performed for each factor assay. Thrombin time was measured using the HemosIL thrombin time kit, and protein S activity was measured using the HemosIL protein S activity kit. Fibrinogen concentrations were measured by the Clauss method using HemosIL Fibrinogen-C reagent. D-dimer (HemosIL D-Dimer 500 kit), von Willebrand factor activity (HemosIL von Willebrand Factor Activity kit), and von Willebrand factor antigen (HemosIL von Willebrand Factor Antigen kit) were measured using turbidimetric immunoassays. Protein C antigen (HemosIL Protein C kit) and alpha-2 plasmin inhibitor (HemosIL Plasmin Inhibitor Kit) were measured using an amidolytic activity assay with a chromogenic substrate. Normal pooled plasma for control measurements was obtained from Precision Biologic (CRYOCheck, Dartmouth, Nova Scotia, Canada).

### Pathogen reduction

Fifteen units of COVID-19 convalescent plasma underwent an automated pathogen reduction process using the US FDA-authorized INTERCEPT Blood System intended for platelet donations (Cerus, Concord, CA, USA), according to manufacturer’s instructions. This system uses a psoralen compound, amotosalen, which binds to nucleic acids and lipids, and intercalates into nucleic acids. Illumination by ultraviolet A light (320–400 nm) leads to the formation of covalent bonds between pyrimidine bases, preventing replication and transcription. Each plasma unit was transferred through an amotosalen container into the INTERCEPT 100 ultraviolet A illuminator for pathogen reduction for 6–10 min. The plasma was then incubated with the compound absorption device of the INTERCEPT system for 12–24 h, shaking at room temperature to remove amotosalen and its photoproducts. All apheresis plasma was frozen within 8 h of collection and all COVID-19 convalescent plasma was frozen within 24 h of collection.

### Statistical analysis

Descriptive statistics are presented as mean ± standard deviation (SD). Separate univariate analyses of variance were used to compare the metrics of the coagulation profile between the three plasma preparations (COVID-19 convalescent plasma, pathogen-reduced convalescent plasma, and fresh frozen plasma). Post hoc analyses using Tukey’s honestly significant difference multiple comparisons were performed to test for differences between pairs of plasma preparations. Assumptions of normality were confirmed with Shapiro–Wilk tests and assumptions of homoscedasticity were confirmed with Levene’s test. Two outliers were identified in the D-dimer assay results, one outlier in both the pathogen reduced COVID-19 convalescent plasma group (4910 ng/dL FEU) and the fresh frozen plasma group (1305 ng/dL FEU). Statistical analyses were performed with removal and inclusion of these outliers, and the interpretation of results did not change. Outliers have been removed from tables and figures.

The interpretation of findings was based on *p* < 0.05. Reported *p* values are two-sided and have been adjusted for multiplicity using Bonferroni factor. Analyses were performed with the use of IBM Statistical Package for Social Sciences version 25 statistical package (Armonk, New York, USA). Figures were prepared using GraphPad Prism software version 9.0.0 (La Jolla, CA, USA).

## Results

### COVID-19 convalescent plasma

**Figure 1:**
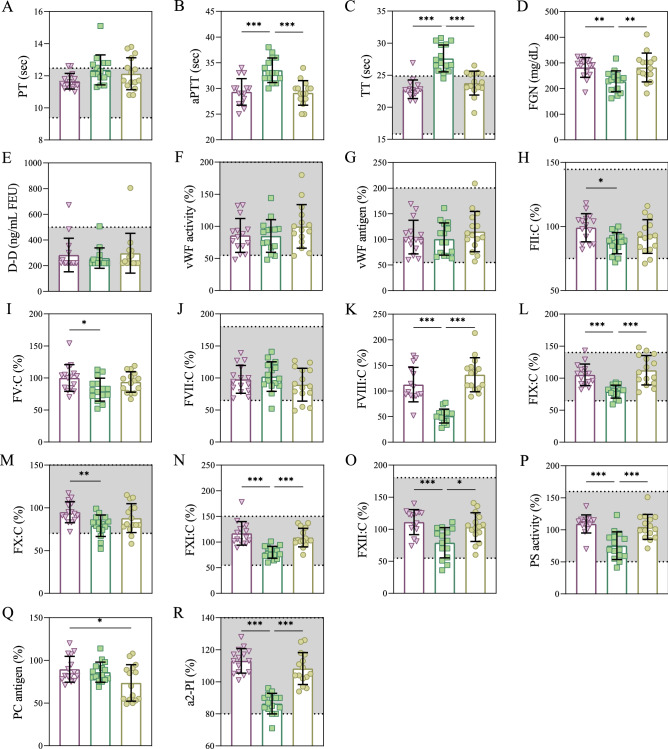
Coagulation profile of COVID-19 convalescent plasma (purple), pathogen reduced COVID-19 convalescent plasma (green), and standard fresh frozen plasma (yellow). The following measurements were made: prothrombin time (PT, s), activated partial thromboplastin time (aPTT, s), thrombin time (TT, s), fibrinogen (FGN, mg/dL), D-Dimer (D-D, ng/dL fibrinogen equivalent units [FEU]), von Willebrand factor (vWF) activity (%), von Willebrand factor antigen (%), various coagulation factors II, V, VII–XII (FII, FV, FVII–FXII, %), protein S (PS) activity (%), protein C (PC) antigen (%), and alpha-2 plasmin inhibitor (a2-PI, %). Individual data are represented as symbols and summary statistics (mean ± standard deviation [SD]) are represented as bar charts. Grouped data are compared using one-way analysis of variance with Tukey’s honestly significant difference (HSD) post hoc test to determine *p* values; *p < 0.05; **p < 0.01; and ***p < 0.001). Horizontal bars above given columns indicate the comparisons being made. Whole blood reference ranges are denoted by grey filled areas bound by dotted black lines. Reference ranges are not available for FGN, FV:C, FVIII:C, and PC antigen.

COVID-19 convalescent plasma contained more protein C antigen than standard fresh frozen plasma but was otherwise not different for any coagulation measures including prothrombin time, activated partial thromboplastin time, thrombin time, fibrinogen, D-dimer, von Willebrand factor activity, von Willebrand factor antigen, coagulation factors II, V, VII–XII, protein S activity, and alpha-2 plasmin inhibitor (Table 2; Fig. 1). These data suggest that the coagulation profile is not different between fresh frozen plasma and COVID-19 convalescent plasma.

**Table 2:** Coagulation profile of three preparations of human plasma, COVID-19 convalescent plasma, pathogen-reduced COVID-19 convalescent plasma, and fresh frozen plasma.

Variable	Convalescent plasma	Pathogen-reduced convalescent plasma	Fresh frozen plasma	*p* values
PT (s)	11.7 ± 0.5	12.4 ± 0.9	12.1 ± 1.0	^ns,^ ^ns,^ ^ns^
aPTT (s)	29.3 ± 2.6	33.5 ± 2.4	29.1 ± 2.4	***^,^^ns,^ ***
TT (s)	22.8 ± 1.4	27.6 ± 2.1	23.8 ± 1.9	***^,^^ns,^ ***
FGN (mg/dL)	282 ± 39	228 ± 41	282 ± 56	***^,^^ns,^ ***
D-D (ng/mL FEU)	283 ± 130	259 ± 79	297 ± 155	^ns,^ ^ns,^ ^ns^
vWF activity (%)	86 ± 26	85 ± 25	100 ± 34	^ns,^ ^ns,^ ^ns^
vWF antigen (%)	104 ± 33	101 ± 31	115 ± 39	^ns,^ ^ns,^ ^ns^
FII:C (%)	99 ± 11	87 ± 8	92 ± 13	*^,^^ns,^ ^ns^
FV:C (%)	100 ± 21	82 ± 18	94 ± 16	*^,^^ns,^ ^ns^
FVII:C (%)	98 ± 22	102 ± 23	90 ± 26	^ns,^ ^ns,^ ^ns^
FVIII:C (%)	113 ± 34	51 ± 14	132 ± 33	***^,^^ns,^ ***
FIX:C (%)	105 ± 17	79 ± 10	112 ± 23	***^,^^ns,^ ***
FX:C (%)	95 ± 12	79 ± 13	88 ± 17	**^,^^ns,^ ^ns^
FXI:C (%)	117 ± 23	80 ± 12	109 ± 18	***^,^^ns,^ ***
FXII:C (%)	111 ± 20	79 ± 24	103 ± 22	***^,^^ns,^ ***
PS activity (%)	109 ± 14	75 ± 22	105 ± 20	***^,^^ns,^ ***
PC antigen (%)	90 ± 15	86 ± 12	74 ± 21	^ns,^ *^,^^ns^
a2-PI (%)	113 ± 8	86 ± 6	108 ± 10	***^,^^ns,^ ***

The following measurements were made: prothrombin time (PT, seconds), activated partial thromboplastin time (aPTT, seconds), thrombin time (TT, seconds), fibrinogen (FGN, mg/dL), D-Dimer (D-D, ng/dL fibrinogen equivalent units [FEU]), von Willebrand factor (vWF) activity (%), von Willebrand factor antigen (%), various coagulation factors II, V, VII–XII (FII:C, FV:C, FVII:C–FXII:C, %), protein S (PS) activity %), protein C (PC) antigen (%), and alpha-2 plasmin inhibitor (a2-PI, %). Data are reported as mean ± standard deviation (SD) and compared using one-way analysis of variance with Tukey’s honestly significant difference (HSD) post hoc test to determine *p* values. *p* values (ns, *p* > 0.05; *p < 0.05; **p < 0.01; and ***p < 0.001) are reported in the order of the following comparisons: convalescent plasma to pathogen-reduced convalescent plasma, convalescent plasma to fresh frozen plasma, and pathogen-reduced convalescent plasma to fresh frozen plasma.

### Pathogen reduction

When compared to fresh frozen plasma and regular convalescent plasma, pathogen reduction treatment prolonged the activated partial thromboplastin time and thrombin time, while also reducing fibrinogen, coagulation factors II, V, VIII, IX, X, XI, XII, protein S activity, and alpha-2 plasmin inhibitor (Table 2; Fig. 1).

## Discussion

### Principal findings

The primary aim of this study was to evaluate the coagulation profile of COVID-19 convalescent plasma in comparison to standard fresh frozen plasma. Consistent with our hypothesis, we found no clinically relevant differences in coagulation profile between COVID-19 convalescent plasma and standard fresh frozen plasma, including key anti-coagulant factors. When comparing pathogen reduction treated convalescent plasma to fresh frozen plasma and regular convalescent plasma, we found prolonged activated partial thromboplastin time, thrombin time, while pathogen reduction treatment reduced fibrinogen, factors II, V, VIII, IX,X, XI, XII, protein S activity, and alpha-2 plasmin inhibitor.

### Coagulation profile of COVID-19 convalescent plasma

Coagulation activation in convalesced COVID-19 patients at four and two and a half months after active disease was previously investigated by von Meijenfeldt et al.^16^ and Fogarty et al.^17^, respectively. Compared to healthy controls, the former found increased levels of factor II, VIII, and plasminogen activator inhibitor-1, whereas the latter reported increased factor II, factor VIII, and von Willebrand factor antigen. In the present investigation, we encountered similar coagulation profiles in COVID-19 convalescent plasma when compared to regular fresh frozen plasma. We analyzed plasma from at least 14-days convalesced donors who previously had laboratory testing confirmed COVID-19, regardless of disease severity and clinical disease course. In the study by von Meijenfeldt et al. all patients had been hospitalized; 74% of patients studied by Fogarty et al. had been hospitalized of whom 22% required intensive care unit admission. More severely ill COVID-19 patients exhibit a more profound coagulation response^18^, which may explain the different findings in these studies.

Interestingly von Meijenfeldt et al.^16^ found increased levels of protein C, like the present study. An increase in protein C antigen may suggest that less activated protein C is being generated in-vivo in patients who recently recovered from COVID-19. This would lead to challenges in regulatory feedback to stop excess thrombin generation and may provide an interesting explanation for the preponderance of thromboembolic phenomena seen in COVID patients and those recovering from COVID. If this hypothesis is true, providing an excess of Protein C antigen by transfusing COVID patients with convalescent plasma may drive the enzyme kinetics towards more activated protein C generation and have a helpful anticoagulant effect.

The findings of the current study are in line with our previous publications on the clinical safety profile of COVID-19 convalescent plasma^2,3^. We found low rates of thrombotic and thromboembolic events related to convalescent plasma transfusion, even in patients who were critically ill from COVID-19^19^. The absence of clinical observations of thrombotic and thromboembolic events related to COVID-19 convalescent plasma transfusion previously reported, combined with the absence of either a heightened procoagulant or depressed anti-coagulant profile of COVID-19 convalescent plasma in the present study, further support the safety of COVID-19 convalescent plasma in the treatment of COVID-19. There is much controversy around the effectiveness of convalescent plasma for COVID-19 with several randomized trials reporting negative findings^20–22^. These trials often provided convalescent plasma to patients late in the disease stage^20,21^ or used plasma with a low antibody titer^21^. Subanalyses from these trials^23^ and data from large observational studies have highlighted that use of high-titer^24–26^ convalescent plasma in patients with low endogenous antibody levels, either because they are early in the disease course^25–27^ or because they are immunodeficient^28^, may provide a survival benefit^29^. Regardless, due to the widespread availability of convalescent plasma compared to other novel therapies, it is plausible that convalescent plasma may continue to be used to treat COVID-19 infection, particularly in resource poor locations^30^.

### Coagulation profile of pathogen reduction treated COVID-19 convalescent plasma

Pathogen reduction treatment using psoralens and ultraviolet A light are known to reduce coagulation factors in plasma^13,14^. Coagulation factors are typically reduced up to 30% depending on the specific factor. Fibrinogen and factor VIII have been reported to be reduced more, while factors II, V, VII, IX, X, XI, XII, protein S, protein C, and alpha-2 plasmin inhibitor are relatively retained^13,14^. Consistent with these previous findings we found relevant reductions in fibrinogen and particularly factor VIII in pathogen reduction treated COVID-19 convalescent plasma in the present study. Amotosalen/ultraviolet A treated plasma is thought to retain enough ‘hemostatic’ function to be used therapeutically for clotting factor replacement^31,32^, but consistent with our findings slight prolongation of coagulation assay times has been reported^14^.

## Limitations

Several limitations resulted from the design of this study. First, we did not attempt to assess coagulation profiles by adding (convalescent) plasma to COVID-19 patient samples. Such endeavors have been reported previously^33^ and have largely focused on investigating samples from patients in active ‘cytokine storm’ whereas the present study is focused on the safety of donated products from recovered patients. Second, we did not evaluate the impact of pathogen reduction treatment on SARS-COV-2 antibody titer levels in the convalescent plasma units. In the context of Ebola virus disease, convalescent plasma that underwent amotosalen and ultraviolet A pathogen reduction treatment did not have reduced IgG antibody titers or neutralizing activity^34^. Third, the assays used to characterize the coagulation profile of COVID-19 convalescent plasma were not comprehensive, and the absence of global hemostatic assays and assays of the fibrinolytic system represent limitations of our approach. We did not assess several key factors associated with hemostatic balance (e.g., antithrombin, plasminogen activators and plasminogen inhibitors), thus, our data should not be used to infer definitive safety of COVID-19 convalescent plasma.

## Conclusion

The coagulation profiles of human COVID-19 convalescent plasma and standard fresh frozen plasma are not different. Pathogen reduction of COVID-19 convalescent plasma reduces coagulation factors and causes a slight prolongation of coagulation times. Our findings do not demonstrate an intrinsic hypercoagulability of convalescent plasma and further support the safety profile of convalescent plasma treatment in the context of COVID-19. A key limitation of the study is that the COVID-19 disease course of the convalesced donors was not characterized.

## Data Availability

Study data cannot be shared publicly because of Institutional Review Board restrictions. Individual participant data underlying the results reported in this publication may be made available to approved investigators for secondary analyses. A scientific committee will review requests for the conduct of protocols approved or determined to be exempt by an Institutional Review Board. Requestors may be required to sign a data use agreement. Data sharing must be compliant with all applicable Mayo Clinic policies. Interested parties may contact the Mayo Clinic Institutional Review Board at uscovidplasma@mayo.edu.
